# Influence of Physical Activity Energy Expenditure on Functional Fitness Among Older Adults

**DOI:** 10.1093/geroni/igaa057.607

**Published:** 2020-12-16

**Authors:** Sally Paulson, Michelle Gray

**Affiliations:** 1 Mount St. Joseph, Cincinnati, Ohio, United States; 2 University of Arkansas, Fayetteville, Arkansas, United States

## Abstract

Remaining physically active as one ages plays a critical role in maintaining health and improving functional capacity. Further, older adults can see additional health-related benefits by increasing intensity, duration, frequency, and/or levels of physical activity. However, there is limited research examining physical activity energy expenditure (PAEE) and measures of functional fitness. Therefore, the purpose was to examine differences between older adults with varying levels of PAEE on selected measures of functional fitness. A sample of 25 adults (age: 74.0±7.1 years) were recruited from an urban area and divided into two groups. PAEE was calculated using the total caloric expenditure per week for all exercise-related activities from a self-reported PA questionnaire. Group one expended less than 3,000 calories per week and group two spent more than 3,000 calories per week performing PA. The selected measures of functional fitness were a 4-m gait speed (GS), 30-s chair stand test (CS-30), 2-min step test (ST), and the 8-foot up and go test (GUG). Data were analyzed using a one-way ANOVA. There was a statistically significant difference between the groups on GS (F1, 24 = 9.29, p < .01) and CS-30 (F1, 24 = 4.37, p = .05). The results yielded a trend for the GUG (p =.06). However, there was not a difference between the groups on the ST (p = .11). These results suggest older adults expending more than 3,000 calories per week performing PA walk faster and have greater lower-body strength.

